# Whole-Genome Sequencing to Identify Missed Rifampicin and Isoniazid Resistance Among Tuberculosis Isolates—Chennai, India, 2013–2016

**DOI:** 10.3389/fmicb.2021.720436

**Published:** 2021-11-22

**Authors:** Sembulingam Tamilzhalagan, Sivakumar Shanmugam, Ashok Selvaraj, Sakthi Suba, Chittibabu Suganthi, Patrick K. Moonan, Diya Surie, Mukesh Kumar Sathyanarayanan, Narayanan Shivaram Gomathi, Lavanya Jayabal, Kuldeep Singh Sachdeva, Sriram Selvaraju, Soumya Swaminathan, Srikanth Prasad Tripathy, Patricia J. Hall, Uma Devi Ranganathan

**Affiliations:** ^1^ICMR-National Institute for Research in Tuberculosis, Chennai, India; ^2^U.S. Centers for Disease Control and Prevention, Atlanta, GA, United States; ^3^Chennai Corporation, Chennai, India; ^4^Central TB Division, Government of India, New Delhi, India; ^5^World Health Organization, Geneva, Switzerland

**Keywords:** drug susceptibility testing, genetic mutations, line probe assay, whole genome sequencing, DR TB

## Abstract

India has a high burden of drug-resistant tuberculosis (DR TB) and many cases go undetected by current drug susceptibility tests (DSTs). This study was conducted to identify rifampicin (RIF) and isoniazid (INH) resistance associated genetic mutations undetected by current clinical diagnostics amongst persons with DR TB in Chennai, India. Retrospectively stored 166 DR TB isolates during 2013–2016 were retrieved and cultured in Löwenstein-Jensen medium. Whole genome sequencing (WGS) and MGIT DST for RIF and INH were performed. Discordant genotypic and phenotypic sensitivity results were repeated for confirmation and the discrepant results considered final. Further, drug resistance-conferring mutations identified through WGS were analyzed for their presence as targets in current WHO-recommended molecular diagnostics. WGS detected additional mutations for rifampicin and isoniazid resistance than WHO-endorsed line probe assays. For RIF, WGS was able to identify an additional 10% (15/146) of *rpoB* mutant isolates associated with borderline rifampicin resistance compared to MGIT DST. WGS could detect additional DR TB cases than commercially available and WHO-endorsed molecular DST tests. WGS results reiterate the importance of the recent WHO revised critical concentrations of current MGIT DST to detect low-level resistance to rifampicin. WGS may help inform effective treatment selection for persons at risk of, or diagnosed with, DR TB.

## Introduction

India contributes 27% of the global burden of drug-resistant tuberculosis (DR TB) (World Health Organization, 2020). Recent data reported to the World Health Organization (WHO) showed the global treatment success rate for multi-drug resistant tuberculosis (MDR TB) is 57% (World Health Organization, 2020). TB diagnostic algorithms prioritize the detection of rifampicin resistance (World Health Organization, 2020). People with isoniazid-resistant TB can be missed in those settings and may not receive the recommended modified treatment regimen (World Health Organization, 2020). Commercially available molecular drug susceptibility tests (DSTs) detect well-characterized mutations associated with antibiotic resistance. The WHO recommends commercially available molecular assays that target well-characterized mutations associated with anti-TB antibiotic resistance in addition to conventional, culture-based phenotypic DST for detection of mutations associated with DR TB (World Health Organization, 2013, 2016, 2018a,b). Despite widespread use of these tests, DR TB may be missed when specific mutations underlying drug resistance are not targeted by commercial assays or well characterized in clinically-resistant *M. tuberculosis* isolates (Campbell et al., 2011; Variava and Martinson, 2018). Additionally, approximately 2–3% of *rpoB* mutations that confer low-level resistance to rifampicin are misclassified as sensitive by phenotypic DST, which led WHO to recommend sequencing be used as the gold standard for RIF resistance detection in 2018 (Miotto et al., 2018; World Health Organization, 2018b, 2021). Several studies have explored the added value of using WGS over the currently available technologies for detecting drug resistance in TB (Bradley et al., 2015; Cohen et al., 2019; Genestet et al., 2020). Whole-genome sequencing (WGS) can detect DR TB resistance-associated mutations, hetero-resistance, and novel mutations, but global implementation has been limited (Brown et al., 2015; Farhat et al., 2016).

In the high-burden setting of Chennai, India, WGS was performed on a convenience sample of retrospective, viable isolates from patients with DR TB to identify mutations associated with rifampicin and isoniazid resistance that may have gone undetected by conventional DSTs. These findings demonstrate that WGS can accurately identify known drug resistance-associated mutations that improves early resistance detection and could be used to guide early, appropriate diagnosis and treatment for patients with DR TB. This study aims to highlight the advantages of WGS in detection of rifampicin and isoniazid resistance over the other available DSTs such as LPA and MGIT.

## Materials and Methods

### Culture Retrieval

A total of 166 viable rifampicin-resistant *M. tuberculosis* isolates stored from patients treated for multidrug-resistant TB in Chennai from 2013 to 2016 were included in this study. Patient isolates with rifampicin resistance, with or without isoniazid resistance results, originally detected by the GenoType MTBDR*plus* line probe assay VER 2.0 (LPA; Hain Life Science GmbH, Baden-Württemberg, Germany) were amplified in Löwenstein-Jensen (LJ) medium prior to study. The clinical strains were handled by well trained staff adhering to all biosafety procedures.

### Phenotypic Drug Susceptibility Testing

Phenotypic DST was performed using mycobacterial growth indicator tubes in BACTEC MGIT 960 system (MGIT; BD, Franklin Lakes, NJ, United States) using the 2018 WHO-recommended critical concentrations (Rifampicin –1.0 μg/ml; isoniazid –0.1 μg/ml) (World Health Organization, 2018a).

### DNA Isolation

Genomic DNA was extracted from LJ-amplified isolates using the CTAB method and purified by the Genomic DNA Clean and Concentrator kit (Zymo Research, Irvine, CA, United States). DNA quality and quantity were measured using NanoDrop and Qubit dsDNA Assay kits (Thermo Fisher Scientific, Waltham, MA, United States).

### Whole-Genome Sequencing

DNA libraries were prepared using NexteraXT DNA Library Preparation and Index kits (Illumina, San Diego, CA). Average library sizes were measured ∼850 bp on the Bioanalyser 2100 System (Agilent Technologies, Santa Clara, CA, United States), normalized in equimolar concentrations and loaded for WGS (MiSeq Reagent Kit v3; Illumina, San Diego, CA, United States). The 2 × 251 cycles of paired-end read sequencing were performed on a Miseq sequencer (Illumina, San Diego, CA, United States). The raw sequence reads were deposited in NCBI Bioproject accession ID: PRJNA741102.^1^

### Data Analysis

After successful quality checks, the FASTQ files were analyzed in CamNIRTResPred, an in-house genomic analytics pipeline (Munir et al., 2019). The raw reads were trimmed using Trimmomatic v0.36 with a minimum base quality of 20. Kraken v1.0 was used to check other microbial contaminations. The reads were mapped to H37Rv reference genome (NC_000916.3) using bwa v0.7.12 and indels mapping correction was done using picard v2.2.4 and GATK v3.5. Samtools v1.3.1 was used to identify variants. Drug resistance detection was done using the database encompassing 444 different mutations of *katG, inhA, ahpC, iniB, ndh, kasA*, and *embA* genes for isoniazid resistance and 163 mutations of *rpoB* gene for rifampicin resistance detection. All these mutations in the database were collected based on previous publications describing the contribution of the mutations for phenotypic drug resistance (Supplementary Table 1). All first-line drug detection based on CamNIRTResPred results were compared with the CRyPTIC database (Allix-Beguec et al., 2018; Supplementary Tables 2, 3). Genotypic and phenotypic DST results were compared; phenotypic and/or genotypic DST were repeated for discordant results. Confirmed discrepancies were further analyzed for the identification of drug-resistance missed by either method.

## Results and Discussion

WGS was performed on 166 isolates collected from 165 patients. Two clinically distinct isolates were obtained 3 years apart from one patient (2013 and 2016). All 166 isolates had rifampicin-resistant LPA results and were classified as MDR TB.

### Rifampicin

Of the 146 rifampicin-resistant isolates, as detected by WGS, 137 (94%) harbored mutations exclusively within the rifampicin-resistance determining region (RRDR) of the *rpoB* gene targeted by LPA probes and were therefore detected as resistant by LPA (Table 1). However, only 131 (90%) were detected as resistant by MGIT DST. Of the MGIT-confirmed rifampicin-resistant isolates, 95/131 (72.5%), 23/131 (18%), and 11/131 (8%) contained the well-characterized, resistance-associated *rpoB* S450L, H445C/D/R/S/Y, and D435G/V/Y mutations, respectively, while the remaining 2/131 (1.5%) harbored resistance-associated mutations at *rpoB* L430P and L452P mutations (Table 1).

**TABLE 1:** Whole-genome sequencing results for 146 isolates harboring mutations associated with rifampicin resistance and their resistance status according to phenotypic (Mycobacterial growth indicator tubes) and MTBDR*plus* line probe assay VER 2.0 drug susceptibility tests.

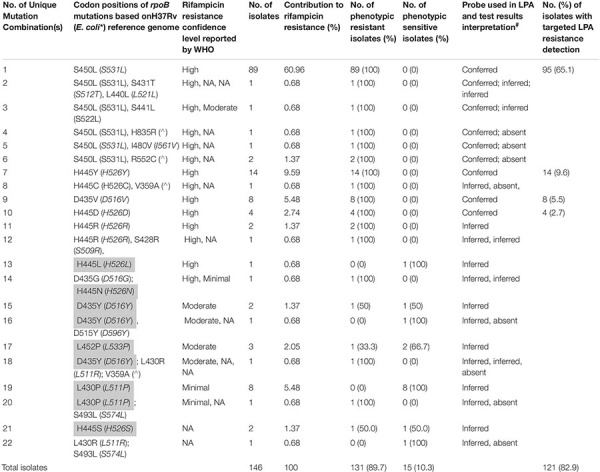

*E.coli genome (given within brackets); ^∧^Not present in the corresponding E. coli genome; Borderline resistance mutations denoted by WHO are shaded in gray (World Health Organization, 2021). ^#^Conferred- LPA interpretation based on mutant probe detection, inferred- LPA interpretation based on lack of wild type probe hybridization, absent- not detected or reported by LPA. Abbreviations: WHO, World Health Organization; NA, not available.

Among the 146 isolates harboring *rpoB* mutations by WGS, nine had *rpoB* mutations outside of the RRDR that have been associated in the literature with RIF resistance [V359A (Walker et al., 2015), I480V (Sandgren et al., 2009; Zhang et al., 2013), S493L (Desjardins et al., 2016; Yang et al., 2017), D515Y (Allix-Beguec et al., 2018), R552C (Sandgren et al., 2009), H835R (Casali et al., 2014)]. Five of these nine (56%) had concurrent RRDR mutations and were resistant by MGIT DST, while 4/9 (44%) had mutations within or outside of the RRDR that are associated with borderline RIF resistance and only 50% were RIF-resistant by MGIT DST. The 18 isolates with WGS-detected mutations harbored *rpo*B L430P (*n* = 8), D435Y (*n* = 4), L452P (*n* = 3), H445S (*n* = 2), H445L (*n* = 1) mutations, which were previously associated with borderline phenotypic RIF resistance at a MGIT RIF DST concentration of 1 μg/ml. All L430P mutants, and two third of L452P mutants and half of D435Y and H445S mutations were phenotypically RIF-susceptible. At the new MGIT RIF DST concentration of 0.5 μg/ml (World Health Organization, 2021) each of these mutations is defined as resistance-associated with the exception of H445S (World Health Organization, 2021). These findings highlight the significant contribution of previously undetected and unreported borderline resistance-associated mutations (12.3%) to RIF-resistant TB in Chennai (Table 1).

In addition to WGS concordance with MGIT DST, 17% of WGS-confirmed *rpoB* mutants are represented as LPA “inferred” based on lack of wild type probe hybridization alone. These findings suggest that inclusion of updated set probes specific to this important and otherwise-undetected set of resistance-associated mutations in future molecular DST assays will aid in improved identification of rifampicin-resistant TB cases (Campbell et al., 2011).

### Isoniazid

Isoniazid resistance related mutations detected by WGS have been previously reported in the coding region of the *kat*G gene and the promoter regions of *inh*A and *ahp*C and are listed in Table 2 (World Health Organization, 2018b). In contrast to rifampicin-resistance results, WGS and MGIT INH DST were 100% concordant (137/137) for INH resistance among Chennai isolates. The majority, or 80.2%, of resistance-associated mutations occurred in *kat*G, followed by 18.2% in *inh*A (coding and promoter regions) and 1.5% in *ahp*C (Table 2).

**TABLE 2:** Whole-genome sequencing mutation analysis of 137 phenotypically isoniazid (INH)-resistant isolates and their detection status using MTBDR*plus* line probe assay VER 2.0.

S. No. of mutation	Mutation (amino acid change)*	WHO INH confidence level of resistance	No. of isolates	Percent contribution to WGS-detected INH resistance	Probe used in LPA and test results interpretation	Percent of potential INH resistance detection by direct LPA probe targeting
1	S315T *katG*	High	88	64.23	Conferred	64.23
2	S315N *katG*	High	5	3.65	Inferred	0
3	S315T *katG*;C-12T *embA*	High, NA	4	2.92	Conferred, absent	2.92
4	S315T_2_ *katG*	High	2	1.46	Conferred	1.46
5	S315T *katG*;C-15T *inhA*	High, Moderate	2	1.46	Conferred, conferred	1.46
6	I21V *inhA*	NA	2	1.46	Absent	0
7	S315T, G297V *katG*	High, NA	1	0.73	Conferred, absent	0.73
8	S315T *katG*;G-17T *inhA*;C-12T *embA*	High, NA, NA	1	0.73	Conferred, inferred, absent	0.73
9	S315T *katG*;C-15T *inhA*;G406A *embB*	High, Moderate, NA	1	0.73	Conferred, conferred, absent	0.73
10	S315N *katG*;F413L*kasA*	High, NA	1	0.73	Inferred, absent	0
11	C-15T *inhA*	Moderate	12	8.76	Conferred	8.76
12	C-15T, I194T *inhA*	Moderate, NA	1	0.73	Conferred, absent	0.73
13	C-15T, I21T *inhA*	Moderate, NA	1	0.73	Conferred, absent	0.73
14	C-15T, I21V *inhA*	Moderate, NA	1	0.73	Conferred, absent	0.73
15	C-15T, S94A *inhA*	Moderate, NA	1	0.73	Conferred, absent	0.73
16	C-15T *inhA*;W191R *katG*	Moderate, NA	1	0.73	Conferred, absent	0.73
17	V1A *katG*	NA	1	0.73	Absent	0
18	W191R *katG*	NA	1	0.73	Absent	0
19	Q295P *katG*	NA	1	0.73	Absent	0
20	W300C *katG*	NA	1	0.73	Absent	0
21	T308P *katG*	NA	1	0.73	Absent	0
22	T-8C *inhA*	NA	1	0.73	Conferred	0.73
23	G-17T *inhA*	NA	1	0.73	Inferred	0
24	I21T *inhA*	NA	1	0.73	Absent	0
25	I194T *inhA*	NA	1	0.73	Absent	0
26	T-8C, I21V *inhA*	NA, NA	1	0.73	Conferred, absent	0.73
27	T-8C *inhA*;W191R *katG*;C-72T *ahpC*	NA, NA, NA	1	0.73	Conferred, absent, absent	0.73
28	G-48A *ahpC*	NA	1	0.73	Absent	0
29	C-81T *ahpC*	NA	1	0.73	Absent	0
All isolates		Total	137	100	–	86.9

*Codon position of mutations based on the Mycobacterium tuberculosis reference strain genome, H37Rv. Conferred- LPA results interpretation based on specific mutant probe, inferred- LPA results interpretation based on non-hybridization with wild type probe, Absent—Neither mutant nor wild type probes available to detect the mutation. *WHO, World Health Organization; NA, not available.*

The high-confidence *kat*G S315T (AGC to ACC codon change) being the most prominent mutation in the cohort, represented in 71% of INH-resistant isolates, the S315T_2_ (AGC to ACA codon change) mutation accounting for 1.5% of all INH resistance (Table 2). *kat*G S315N (AGC to AAC codon change) accounted for an additional 4% of INH resistance and was recently highlighted by WHO for its contribution to high-level isoniazid resistance (World Health Organization, 2018b).

Importantly, this S315N *katG* mutation (Boonaiam et al., 2010; Feuerriegel et al., 2015; Walker et al., 2015) was present in 6 of the isolates with INH resistance determined by WGS and MGIT DST that would have had resistance only “inferred” by LPA based on the absence of wild type and mutant bands. Other WGS-detected mutations that were similarly undetectable by LPA included *kat*G V1A (Sandgren et al., 2009), W191R (Sandgren et al., 2009; Walker et al., 2015), Q295P (Sandgren et al., 2009), W300C (Feuerriegel et al., 2015; Walker et al., 2015), and T308P (Sandgren et al., 2009); *inh*A G-17T (Desjardins et al., 2016), I21V (Boonaiam et al., 2010), I21T (Sandgren et al., 2009; Walker et al., 2015) and I194T (Sandgren et al., 2009; Feuerriegel et al., 2015; Walker et al., 2015); and *ahp*C C-81T (Slayden and Barry, 2000) and G-48A (Slayden and Barry, 2000; Jnawali et al., 2013; Table 2). These mutations were identified in 18/137 (13%) phenotypically-confirmed isoniazid resistant isolates, making them ideal additions to future molecular sensitivity tests for INH (Table 2). The other major TB drugs such as ethambutol, pyrazinamide, streptomycin, aminoglycosides, fluoroquinolones, and ethionamide resistance associated mutations were also detected in these 166 isolates by WGS and were compared with their phenotypic DST (MGIT) results (Supplementary Table 4).

### Drug-Resistant Tuberculosis Pattern Detection by Whole-Genome Sequencing and MGIT Drug Susceptibility Test on the LPA Identified Multi-Drug Resistant Tuberculosis Patients

Of the 165 LPA identified MDR TB patient, WGS and MGIT DST identified the presence of pan-drug susceptibility in 8 and 6%; mono-isoniazid resistance in 2.4 and 6.7%; DR other than mono-INH and MDR resistance in 1.8 and 7.3% of patients, respectively. The MDR TB patient numbers were reduced to 12 and 20%, respectively, as verified by WGS and MGIT DST results (Table 3). Based on our results comparison, WGS identified 20 (12%) false-positive cases detected by LPA. This limitation of LPA with 93.8% specificity for rifampicin resistance detection was previously reported (Rufai et al., 2014; Ninan et al., 2016). The whole genome sequencing in Miseq platform costs approximately 18,000 Indian rupees per sample at our facility. As the LPA kits were received at a subsidized cost, we are unable to perform a cost comparison among the molecular diagnostics in an ideal scenario. However, a recent study from South Korea analyzed the costs associated with various methodologies for DST (Kim et al., 2019). A similar analysis of associated costs in India may inform policies related to the appropriate application of these methodologies for the detection of drug-resistance in clinical, programmatic and research settings.

**TABLE 3:** Resistance pattern concordance by WGS and MGIT DST among the LPA identified MDR TB patients.

Resistance category	WGS result No. of patients (% change*)	MGIT result No. of patients (% change*)
**Susceptible**	13 (+ 8%)	10 (+ 6%)
**Mono-INH**	4 (+ 2.4%)	11 (+ 6.7%)
**MDR TB**	145 (–12%)	132 (–20%)
**^∧^Other drug resistance**	3 (+ 1.8%)	12 (+ 7.3%)
**Total**	165	165

*Compared with LPA result. ^∧^Drug resistance identified other than Mono-INH and MDR TB.

## Conclusion

Commercially available phenotypic and genotypic DSTs do not comprehensively detect patients with DR TB. In our study, WGS identified known mutations associated with TB resistance to RIF and INH not detected by other methods. Given that study isolates were preselected for LPA-determined drug resistance, future studies should include isolates detected by other WHO-recommended, rapid molecular diagnostic tests and/or LPA-determined sensitive strains to better quantify the extent of undetected drug resistance in the population. As sequencing-based technologies become simpler and more affordable, whole-genome and/or targeted sequencing approaches are likely to become preferred options for early and accurate detection of drug-resistant TB. These data confirm the 2021 WHO revision of the critical concentration for rifampicin MGIT DST and further underscore the important contribution of borderline and low-level resistance detection to DR TB. Given the proportions of potentially false rifampicin resistance and undetected isoniazid-resistance by LPA in this study, testing for all resistance-associated and/or novel mutations by WGS among persons at risk of drug-resistant TB would best inform patient classification and treatment in India.

## Data Availability Statement

The datasets presented in this study can be found in online repositories. The names of the repository/repositories and accession number(s) can be found below: https://www.ncbi.nlm.nih.gov/, PRJNA741102.

## Ethics Statement

This study was approved by the US Centers for Disease Control and Prevention, Center for Global Health (Human Subject Research Tracking No. 2017-461); the Ethical Committee of ICMR National Institute for Research in Tuberculosis, Chennai (Institutional Ethics Committee No. 2015019); Indian Council of Medical Research; and the Revised National Tuberculosis Control Programme, Indian Ministry of Health and Family Welfare. Informed consent was waived because this involved only routine samples collected for the National TB Elimination Program (NTEP).

## Author Contributions

STa and UR: study conception and design. STa, SSh, AS, SSu, and CS: acquisition of data. NG, LJ, KS, SSe, and STr: clinical resources. STa, SSh, AS, PM, DS, PH, MS, and CS: analysis and interpretation of data. STa, PM, DS, PH, SSw, STr, and UR: drafting the article and revising for intellectual content. UR: had full access to all of the data in the study and took responsibility for the integrity of the data and the accuracy of the data analysis. All authors were involved in drafting the article or revising it critically for important intellectual content, and approved the final version to be published.

## Author Disclaimer

The findings and conclusions in this report are those of the authors and do not necessarily represent the official position of the US Centers for Disease Control and Prevention and other funding institutions.

## Conflict of Interest

The authors declare that the research was conducted in the absence of any commercial or financial relationships that could be construed as a potential conflict of interest.

## Publisher’s Note

All claims expressed in this article are solely those of the authors and do not necessarily represent those of their affiliated organizations, or those of the publisher, the editors and the reviewers. Any product that may be evaluated in this article, or claim that may be made by its manufacturer, is not guaranteed or endorsed by the publisher.

## Footnotes

1
https://www.ncbi.nlm.nih.gov/bioproject

## Supplementary Material

A supplementary file encompassing mutation database used in the analytic pipeline, a completely analyzed WGS data for 166 isolates using the in-house mutation database as well as cryptic database and the sensitivity and specificity comparison of these two mutation databases with reference to phenotypic DST were given separately as MS-Excel.
